# Insecticide Filtration Efficiency of Respiratory Protective Equipment Commonly Worn by Farmers in Thailand

**DOI:** 10.3390/ijerph18052624

**Published:** 2021-03-05

**Authors:** Ratana Sapbamrer, Surat Hongsibsong, Manoch Naksata, Wimol Naksata

**Affiliations:** 1 Department of Community Medicine, Faculty of Medicine, Chiang Mai University, 110 Inthavaroros Road, Sri Phum Subdistrict, Muang District, Chiang Mai 50200, Thailand; 2 Environmental and Occupational Health Sciences and Non Communicable Diseases Center of Excellence, Chiang Mai University, 110 Inthavaroros Road, Sriphum Subdistrict, Muang District, Chiang Mai 50200, Thailand; 3 School of Health Sciences Research, Research Institute for Health Sciences, Chiang Mai University, 110 Inthavaroros Road, Sriphum Subdistrict, Muang District, Chiang Mai 50200, Thailand; 4 Department of Physics and Material Science, Faculty of Science, Chiang Mai University, 239, Huay Kaew Road, Suthep Subdistrict, Muang District, Chiang Mai 50200, Thailand; manochnak@gmail.com (M.N.); wimol.n@cmu.ac.th (W.N.)

**Keywords:** pesticide, insecticide, respiratory protective equipment, mask, farmer, occupational exposure, inhalation

## Abstract

Farmers are at a high risk of inhalation exposure when handling pesticides. Thai farmers usually protect themselves against pesticide exposure by wearing commercial respiratory protective equipment (RPE) available from rural community markets. However, scientific data regarding the pesticide filtration efficiency of RPE commonly worn by farmers is limited. Thus, this study aimed to investigate the efficiency of insecticide filtration of various RPE commonly worn by farmers in Thailand. The half facepiece respirator was used as a control to compare the results with other RPE. Ten types of RPE were selected for testing. The filtration efficiency of each RPE against insecticides was tested in a laboratory. The remarkable findings were that a surgical mask demonstrated the least filtration efficiency of all tested insecticides, with a range of 25.7–61.5%. The RPE available in rural markets of Thailand had a filtration efficiency within a range of 64.9–95.4%, whereas a half facepiece respirator was the most efficient in filtering insecticides, with a range of 96.5–98.9%. Therefore, our results suggest that the RPE most frequently worn by farmers may not provide adequate protection when compared with the respirator. However, considerations around RPE use in low-and middle-income countries and tropical climate conditions should be based on pesticide toxicity and practical use, ensuring balance between the risks from pesticide exposure and acceptance of PPE use.

## 1. Introduction

Pesticides are substances that are widely used in agriculture for crop protection and in public health to control vector-borne infectious diseases. Organophosphates and pyrethroids are classified as insecticides, and these insecticides are the most widely used in agricultural and public health sectors [1]. Chemicals in the form of particulates, vapors, gasses, and mists have a high potential for inhalation exposure, and cause serious damage to nose, throat, and lung tissues [1,2,3]. Epidemiological studies available for investigation have shown that exposure to insecticides is associated with acute respiratory health effects, including coughing, wheezing, phlegm, breathlessness, chest pain, dyspnea, and nasal irritation. Exposure to insecticides has also been linked to chronic respiratory health effects, including asthma, chronic obstructive pulmonary disease, and chronic bronchitis [4,5].

Respiratory protective equipment (RPE) is used to protect against pesticide inhalation and subsequent absorption through the respiratory system. Wearing appropriate RPE when handling pesticides can minimize damaging exposure and reduce the risks of adverse respiratory symptoms and related diseases. Since several pesticides are classified as organic vapors and pesticide spraying through nozzles produces fine particles, the Food and Agriculture Organization of the United Nations (FAO) and World Health Organization (WHO) recommend that pesticide handlers should wear at least a respirator during handling of pesticides [6]. The United States Environmental Protection Agency (US EPA) has also published the 2015 revised Workers Protection Standards for agricultural pesticide use and recommended that the minimum requirement of RPE when handling pesticides in toxicity category I (extremely and highly hazardous) is a respirator with particulate filter. Nuisance dust masks and surgical masks are not recommended [6,7]. In fact, the RPE worn by pesticide handlers in developing countries did not meet the criteria for pesticide protection. In addition, a systematic review by Sapbamrer and Thammachai [8] found that only 43.2% and 13.9% of pesticides handlers across the world wore masks and respirators respectively, whilst working with pesticides. Unfortunately, most farmers wore RPE made of fabric which would not protect against pesticides efficiently. The factors contributing to the non-compliance with RPE recommendations by farmers included financial problems, availability, and thermal and mechanical discomfort [9]. There was hardly any evidence to indicate useof a respirator due to the lack of affordability.

Thailand is one of the world’s exporters of commodity crops. Most farms are classified as small-scale farming and are operated by family members. Approximately 38% of the Thai population work in the agricultural sector; however, agricultural Gross Domestic Product (GDP) was only 12% [10,11,12]. Importantly, Thai farmers are still facing financial problems and the level of education in the agricultural system is low [12,13]. At the start of this study, it was found that most farmers had a low level of pesticide knowledge and showed a lack of safety awareness [14,15,16]. Regarding the use of RPE, wearing a mask during pesticide application ranged from 12.2% to 88.4% [14,17,18,19,20]. Significantly, the farmers usually use masks which are available in the markets within their community which are made with fabric. Despite concerns, available studies which evaluate the pesticide filtration efficiency of RPE commonly worn by farmers are limited. Thus, the aim of this study was to investigate the insecticide filtration efficiency of the various types of respiratory protective equipment commonly worn by farmers in developing countries, which included Thailand, and to compare the filtration efficiency of the RPE with a half facepiece respirator. The half facepiece respirator was used as a control to compare the results with the other types of RPE. The insecticides used in the tests were selected as they are extensively imported into Thailand. They included chlorpyrifos, profenofos, omethoate, diazinon, cypermethrin, and deltamethrin [21].

## 2. Materials and Methods

### 2.1. Tested RPE

Ten examples of RPE were selected for testing. Seven types of the RPE were commonly used for pesticide protection by Thai farmers, including surgical mask, sun hat, ‘robber mask’ with woven fabric, robber mask with knitted fabric, activated carbon mask, cotton mask, and bandana (see Table 1). Three types of RPE were respirators conforming to FAO and WHO recommendations, including a half facepiece respirator (3M-7502 with cartridge filter number 60926, P100), an organic vapor respirator without valve (3M-8247, R95), and an organic vapor respirator with valve (3M-9913V, GP). These two organic vapor respirators can protect against pollutants which are in the form of dust and gasses. The results from the half facepiece respirator were used as a control for comparison with the other RPE. The cartridge filter number 60926 is classified as NIOSH-approved P100, implying that the filtration level is at least 99.97% of airborne particles, and strongly resistant to oil. The respirator of 3M-8247 is classified as NIOSH-approved R95, implying that the filtration level is at least 95% of airborne particles, and somewhat resistant to oil. The respirator of 3M-9913V is classified as AS/NZS 1716 Standard-approved GP1, implying that filtration level is at least 80% of airborne particles, and suitable for organic compounds.

The surgical masks and activated carbon masks were purchased from a pharmacy. The sun hat, robber mask with woven fabric, robber mask with knitted fabric, cotton mask, and bandana were samples from rural markets in Thailand. The respirators and half facepiece respirator were purchased from a 3M distributor in Thailand. Table 1 shows the information and image for each of the RPE types, including material type, the number of layers, thickness, available source, price, and other pertinent information.

### 2.2. Tested Insecticides

Six insecticides were selected for this study. These were chlorpyrifos 40% w/v, emulsifiable concentrate (Comic40, ICP Ladda Co., Ltd., Bangkok, Thailand); profenofos 50% w/v, emulsifiable concentrate (Thanyatip Chemical Co., Ltd., Bangkok, Thailand); omethoate 50% w/v soluble concentrate (Modern, ICP Ladda Co., Ltd., Bangkok, Thailand); diazinon 60% w/v emulsifiable concentrate (Diazinon 60, Kerakon Co., Ltd., Bangkok, Thailand); cypermethrin 35% w/v emulsifi-able concentrate (Thaiperthroid 35, Pato Chemical Co., Ltd., Bangkok, Thailand); deltamethrin 3% w/v emulsifiable concentrate (S&P Formulator Co., Ltd., Bangkok, Thailand). These insecticides were in the top ten chemicals imported into Thailand [21]. The insecticides were diluted to a concentration of 2 mL/1 L of water, which was in accordance with the recommendation of the dilution on the labels of insecticides.

### 2.3. Experimental Design

**Figure 1:**
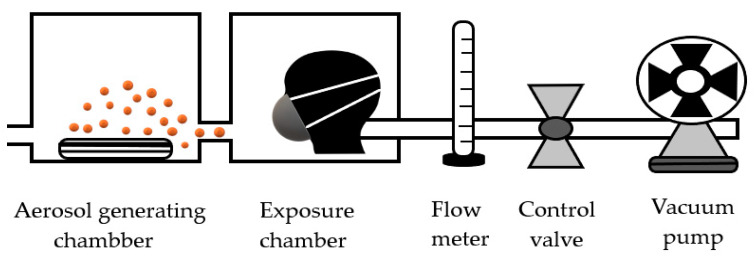
Experimental setup.

The filtration efficiency of the tested RPE was tested in closed chambers, methods modified from the ISO 16900-3 standard [22] and the study by Shakya et al. [23]. The experimental design is presented in Figure 1. The experimental apparatus used consisted of an aerosol generating chamber and an exposure chamber. These two chambers were connected with a tube of diameter 1 inch and 1 inch in length. Pesticide aerosol was generated in the aerosol generating chamber (0.6 m × 0.40 m × 0.40 m). The aerosol was generated by using an ultrasonic atomizer (Leifen, Guangdong, China), which had a production capacity of 5000 mL/h, and produced a median droplet size of 5.6 microns [24]. Then, the aerosol was directed to the exposure chamber (0.6 m × 0.40 m × 0.40 m), which had a mannequin head inside. A hole of diameter 1 inch was made in the nose of the mannequin for connection with a vacuum pump (Daikawa#2VP-250L, Japan). The mannequin head was fitted with each RPE tested, and a 10 cm diameter pad of alpha cellulose was placed between the RPE and the mannequin for measuring pesticide concentrations through the tested RPE. During the experiment, the RPE was attached to the mannequin with silicone tape for prevention of a leakage. As a control, the pad was also attached to the nose zone of the mannequin without RPE for measuring actual pesticide concentrations. Pesticide concentration in the pad from the mannequin with and without RPE was measured. The concentration in the pad from the mannequin with RPE is representative of the amount of pesticides that passes through the tested RPE. The concentration in the pad from the mannequin without RPE is representative of total amount of pesticides that come into the respiratory system. The test was conducted at a flow rate of 90 mL/min through each RPE, a rate representative of moderate exertion during physical activity [25,26]. A suction pump was used to create and maintain air flow, and a flow meter was used to control the flow rate as required, according to the ISO 16900-3 standard [22]. The experimental duration was set at 30 min because the survey of Laird et al. [27] stated that the task duration for agricultural chemical spraying was approximately 20–30 min. At the end of the experiment, the pad from the mannequin with and without RPE was collected, and the concentrations of the insecticides were analyzed. The procedure was repeated 5 times with each RPE tested. The test on the mannequin without RPE was also repeated 5 times. To control the quality of laboratory testing in each batch, the test from the mannequin without RPE was conducted before testing the pad from the mannequin with RPE. All pad samples were kept in a freezer at −20 °C before pesticide analysis.

### 2.4. Extraction and Analysis of Insecticides

Six insecticides, including chlorpyrifos (CAS Number: 2921-88-2), profenofos (CAS number: 41198-08-7), omethoate (CAS number: 1113-02-6), cypermethrin (CAS number: 52315-07-8), and deltamethrin (CAS number: 52918-63-5) were purchased from Dr. Ehrenstorfer GmbH (Augsberg, Germany). The alpha cellulose pad samples were extracted and analyzed by using a modification of the method described by Sapbamrer and Hongsibsong [28] and Pakvilai et al. [29]. The pad sample was extracted using 20 mL of acetonitrile (HPLC grade, J.T. Baker, Phillipsburg, NJ, USA), and was shaken for 5 min. The extraction was repeated twice with 20 mL and 10 mL of acetonitrile, respectively. Three g of magnesium sulfate (analytical grade, Fluka, Buchs, Germany) and sodium chloride (analytical grade, Fluka, Buchs, Germany) were added to the extract solution to remove water. The solution was filtered through filter paper, which had 2 g of anhydrous sodium sulfate (analytical grade, Fluka, Buchs, Germany), and was then evaporated until dry using a rotary evaporator at 40 °C. The evaporation flask was rinsed with 5 mL of ethyl acetate (HPLC grade, J.T. Baker, Phillipsburg, NJ, USA), and dried with nitrogen. Finally, the residue was reconstituted in 1 mL of ethyl acetate through a 0.25 μm syringe filter.

Organophosphate insecticides, including chlorpyrifos, profenofos, omethoate, and diazinon, were analyzed using gas chromatography (Hewlett-Packard 7890 Series, Palo Alto, CA, USA) equipped with a flame photometric detector (FPD) and a capillary column DB-1701 (14%cyanopropyl-phenyl-methylpolysiloxane column-0.25 mm. I.D. × 30 m length × 0.25 μm film thickness). The temperature was set at 250 °C for injection port (spitless mode) and 250 °C for detection port. Pyrethroid insecticides, including cypermethrin and deltamethrin, were analyzed using a gas chromatograph (Hewlett-Packard 7890 Series, Palo Alto, CA, USA) equipped with an electron capture detector (ECD) and a capillary column HP-5 (5% phenyl-methylpolysiloxane nonpolar column-0.25 mm. I.D. × 30 m length × 0.25 μm film thickness). The temperature was set at 250 °C for injection port (spitless mode) and 300 °C for detection port. Total run time was 50 min, and helium 99.999% at 1.5 mL/min was used as the carrier gas.

### 2.5. Quality Control

The quality control values of the tested insecticides are shown in Table 2. The limitation of detection (LOD) ranged from 0.001 μg for diazinon and deltamethrin to 0.1 μg for omethoate, and the limit of quantification (LOQ) ranged from 0.01 μg for diazinon and deltamethrin to 0.5 μg for omethoate. Recoveries ranged from 87.6% for deltamethrin to 110.6% for chlorpyrifos and cypermethrin.

### 2.6. Data Analysis

The percentage of filtration efficiency was calculated using the following formula:

Filtration efficiency (%) = ((Cno_RPE_ − C_RPE_) × 100)/Cno_RPE_

where:

C_noRPE_ = pesticide concentrations in the pad from mannequin without RPE

C_RPE_ = pesticide concentration in the pad from mannequin with RPE

Average values of insecticide filtration efficiency are presented as mean, median, and standard deviation (SD.). The data are non-normally distributed and therefore the Mann–Whitney U test was used to test the comparison of the filtration efficiency of tested RPE with a half facepiece respirator. *p* value < 0.05 was statistically significant.

## 3. Results

### 3.1. Insecticide Concentrations in the Pad from Mannequin with and without RPE

The lowest concentration of insecticides was found in the pad from the mannequin with a half facepiece respirator (0.012 mg/mL for chlorpyrifos, 0.009 mg/mL for profenofos, 0.109 mg/mL for omethoate, 0.028 mg/mL for diazinon, 0.009 mg/mL for cy-permethrin, and 0.0011 mg/mL for deltamethrin). The highest concentration of insecticides was found in the pad from the mannequin with a surgical mask (0.261 mg/mL for chlorpyrifos, 0.258 mg/mL for profenofos, 1.500 mg/mL for omethoate, 1.310 mg/mL for diazinon, 0.203 mg/mL for cypermethrin, and 0.0127 mg/mL for deltamethrin). The con-centration in the pad from the mannequin without RPE was 0.581 mg/mL for chlorpyrifos, 0.405 mg/mL for profenofos, 2.22 mg/mL for omethoate, 2.37 mg/mL for diazinon, 0.523 mg/mL for cypermethrin, and 0.0349 mg/mL for deltamethrin (Table 3).

### 3.2. Comparison of Insecticide Filtration Efficiency of Various RPE with Half Facepiece Respirator

**Figure 2:**
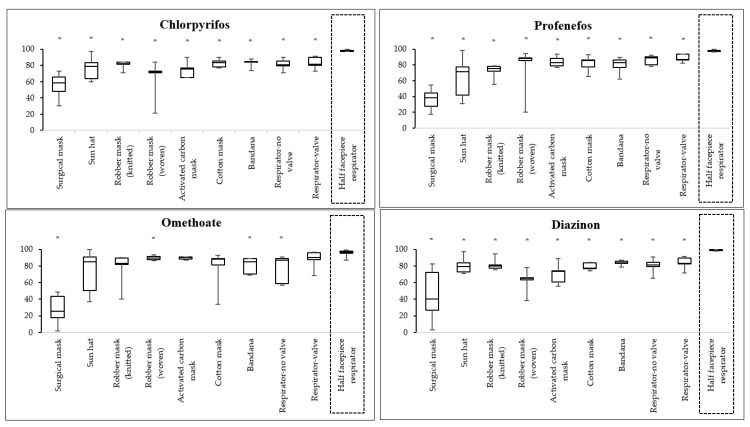
Percentage of organophosphate filtration efficiency of various RPE compared with the half facepiece respirator. * percentages of filtration efficiency for tested RPE were significantly different to that of the half facepiece respirator (*p* < 0.05).

**Figure 3:**
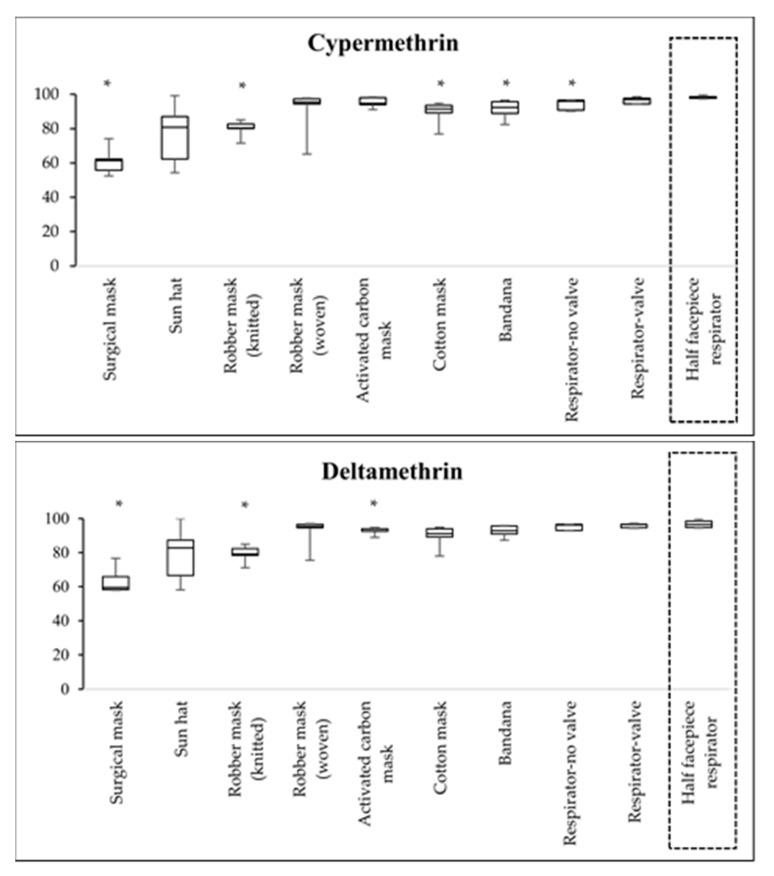
Percentage of pyrethroid filtration efficiency of various RPE compared with the half facepiece respirator. * percentages of filtration efficiency for tested RPE were significantly different to that of the half facepiece respirator (*p* < 0.05).

Insecticide filtration efficiency of various RPE compared with the half facepiece respirator is presented in Figure 2 and Figure 3.

Chlorpyrifos: The surgical mask had the lowest filtration efficiency of chlorpyrifos (58.7%), followed by the robber mask with woven fabric (72.3%), activated carbon mask (75.4%), and sun hat (78.6%), while the half facepiece respirator had the highest filtration efficiency (97.7%). When comparing the filtration efficiency of various RPE with the half facepiece respirator, the filtration efficiencies of all RPE were statistically significantly lower than those of the half facepiece respirator (*p* < 0.05). 

Profenofos: The surgical mask had the lowest filtration efficiency of profenofos (38.4%), followed by the sun hat (71.4%), robber mask with knitted fabric (75.2%), and activated carbon mask (82.6%), while the half facepiece respirator had the highest filtration efficiency (97.3%). When comparing the filtration efficiency of various RPE with the half facepiece respirator, the filtration efficiencies of all RPE were statistically significantly lower than those of the half facepiece respirator (*p* < 0.05).

Omethoate: The surgical mask had the lowest filtration efficiency of omethoate (25.7%), followed by the robber mask with knitted fabric (82.8%), sun hat (85.2%), and bandana (85.3%), while the half facepiece respirator had the highest filtration efficiency (96.5%). When comparing the filtration efficiency of various RPE with the half facepiece respirator, the filtration efficiencies of the surgical mask, robber mask with woven fabric, bandana, and organic vapor respirator without valve were significantly lower than those of the half facepiece respirator (*p* < 0.05).

Diazinon: The surgical mask had the lowest filtration efficiency of diazinon (40%), followed by the robber mask with woven fabric (64.9%), activated carbon mask (73.2%), and cotton mask (77.6%), while the half facepiece respirator had the highest filtration efficiency (98.9%). When comparing the filtration efficiency of various RPE with the half facepiece respirator, the filtration efficiencies of all RPE were significantly lower than those of the half facepiece respirator (*p* < 0.05).

Cypermethrin: The surgical mask had the lowest filtration efficiency of cypermethrin (61.5%), followed by the robber mask with knitted fabric (80.4%), sun hat (80.8%), and cotton mask (91.5%), while the half facepiece respirator had the highest filtration efficiency (98.1%). When comparing the filtration efficiency of various RPE with the half facepiece respirator, the filtration efficiencies of the surgical mask, robber mask with knitted fabric, cotton mask, bandana, and organic vapor respirator without valve were significantly lower than those of the half facepiece respirator (*p* < 0.05).

Deltamethrin: The surgical mask had the lowest filtration efficiency of deltamethrin (59.4%), followed by the robber mask with knitted fabric (79.2%), sun hat (82.8%), and cotton mask (91.2%), while the organic vapor respirator had the highest filtration efficiency (96.6%). When comparing the filtration efficiency of various RPE with the half facepiece respirator, the filtration efficiencies of the surgical mask, robber mask with knitted fabric, and activated carbon mask were significantly lower than those of the half facepiece respirator (*p* < 0.05).

## 4. Discussion

Of the ten types of RPE tested, the surgical mask demonstrated the least filtration efficiency for all tested insecticides (ranged from 25.7% to 61.5%). The surgical mask is designed to reduce exposure to disease transmission by body fluids, such as blood, droplets, and splashes. It is also designed to prevent the body fluids from the wearers releasing to others. Therefore, the surgical mask is intended to be worn by healthcare workers and infected persons [6,30]. It can provide protection from only large spray aerosols and non-hazardous for health. Since insecticides are hazardous chemicals and spraying insecticides through nozzles produces fine aerosols, a surgical mask cannot protect against insecticides efficiently, leading to large amounts of insecticides passing through the surgical mask and being inhaled into the respiratory system. Regarding the activated carbon mask, the filtration efficiency of tested insecticides ranged from 73.2% to 94.8%. When comparing its filtration efficiency with the surgical mask, the efficiency of the activated carbon mask was higher than the surgical mask in all tested insecticides. Activated carbon is most common adsorbent due to its large volume of micropores, mesopores, and large internal surface area. Therefore, these results can be suggested as due to the activated carbon layer in the activated carbon mask acting as an adsorbent to adsorb toxic gasses and vapors, which included insecticides [31,32]. In addition, activated carbon also has anti-microbial and odor reduction properties [33].

Our results showed that the half facepiece respirator was the most efficient in the filtration of insecticides (a range of 96.5% to 98.9%). Our results are in agreement with the study by Penconek et al. [34] which reported that the filtration efficiency of commercially available half facepiece respirators was 75–89% when it came to filtering out diesel exhaust particles. They also suggested that the protection level of commercially available half facepiece respirators may not be sufficient to protect against inhalation of diesel exhaust particles. The half facepiece respirator with cartridge filter used in this study is classified as a NIOSH-approved P100 and is recommended by FAO and WHO, meaning that its minimum filtration efficiency is 99.97% of airborne particles. The standard test is con-ducted using sodium chloride or dispersed oil particles. Our study indicated that insecticides were more likely to penetrate through the filter than standard salt or oil test aerosols. In addition, the actual efficiency of the half facepiece respirator in the standard test may be over-estimated for several reasons [9]. Several factors including face size and shape, facial characteristics, movement, work rate, and wearing time, should be considered [6]. Therefore, respirator fit testing during actual situations involving movement should be done to ensure that the respirator is a close fit for the face of the wearer resulting in an adequate face seal. The study by Føreland et al. [35] reported that the pass rate for all adequacy of fit tests of respirators was 62%, the silicon respirator having the highest pass rate (92–100%). Our study did not investigate the efficiency of insecticide filtration during situations involving movement and further work is warranted in this area.

Our results also showed that organic vapor respirators with and without valves had an efficiency of insecticide filtration inferior to that of the half facepiece respirator. These respirators can protect against pollutants in the form of gasses, vapors, and particulate matter. The FAO and WHO recommend that pesticide handlers should wear a minimum of a respirator during handling of pesticides. Although the FAO and WHO recommend it, our results found that the organophosphate filtration efficiencies of most tested insecticides (including chlorpyrifos, profenofos, diazinon, and cypermethrin) of organic vapor respirators both with and without valves were significantly lower than a half facepiece respirator. These respirators are designed to protect against particulates in the form of solids and liquids with a particle size more than 0.3 microns [6]. Therefore, the fine aerosols produced during the spraying of insecticides might be not filtered efficiently by the filters of the organic vapor respirators. When comparing the efficiencies of insecticide filtration between vapor respirators with and without valves, the efficiencies were rather similar. Exhalation valves are designed to ventilate against heat, humidity, and carbon dioxide within the space of the respirator, and decrease exhalation resistance [36]. However, the exhalation valve is a vulnerable part of the respirator. Damage to the exhalation valve during working conditions may cause higher inward leakage [37].

Although the half facepiece respirator and the organic vapor respirator were the most efficient as regards the filtration of insecticides, decisions to use RPE should also be based on the toxicity of pesticides. The United States Environmental Protection Agency (US.EPA) has published the 2015 revised Workers Protection Standards for agricultural pesticide use and recommended that the minimum requirement of RPE when handling pesticides in toxicity category I (extremely and highly toxic hazardous) is a respirator with a particulate filter. They also recommended these for use during the application of pesticides in high concentrations, with a very fine aerosol, and in enclosed areas [6,7]. Therefore, the use of the respirator is necessary only in situations of high-risk exposure and health. Significantly, these respirators are of relatively high cost, and farmers in low- and middle-income countries have no purchasing power to buy the RPE strongly recommended on the pesticide labels. Many workers also have limited education and low levels of literacy preventing complete understanding of the label instructions. Another factor is that wearing the respirators in tropical climates under hot and humid conditions makes the wearers feel uncomfortable, leading to heat stress and dehydration, resulting in un-acceptance of RPE use. For all these reasons, the gap between RPE requirements as per the label instructions and suitable RPE equipment for farmers in low- and middle-income countries and tropical climates should be considered in maintaining the balance between the risks from pesticide exposure and acceptance of RPE use [6,8,13].

Of the five types of RPE which were available in rural markets of Thailand (including the sun hat, robber mask with woven fabric, robber mask with knitted fabric, cotton mask, and bandana), all had organophosphate filtration efficiency, apart from against omethoate, at a significantly lower efficiency than the half facepiece respirator. However, the results pertaining to pyrethroid filtration efficiency were rather varied. Some of the RPE had pyrethroid filtration efficiency at a significantly lower efficiency than the half facepiece respirator. Previous studies investigating the pesticide filtration efficiency of cloth masks are limited, but those that are available investigated the filtration efficiency of cloth masks to protect against particulate matter. When comparing our results with similar previous studies, our results are in agreement with the study by Shakya et al. [23] which mentioned that commercially available fabric masks had the least filtration efficiency of 39–65% of particulate matter (PM). The study by Mueller et al. [38] also found that the filtration efficiency of cloth masks in the case of protection against volcanic ash ranged between 17.5% and 75%. The study by Pacitto et al. [39] also showed the effectiveness of commercial face masks for PM2.5 in the range 14–96%. In our study, these RPE were inexpensive, easily available, and reusable; therefore, they were a popular choice for Thai farmers. They believed that these pieces of RPE gave them sufficient protection against pesticide exposure. The most common RPE used were made of cotton fabric. Cotton fabric is widely used because of its porosity and hydrophilic properties, resulting in the wearer feeling comfortable and being able to breathe easily [40]. However, these types of RPE made of cotton fabric gave limited protection against pesticides, some penetrating through the pores of the RPE and subsequently entering the respiratory system. In general, several factors affected the penetration of the pesticides through the woven fabrics including type of fabric, thickness and weight of fabric, and pore size of fabric [41,42].

If we consider filling the gap between RPE requirements and suitable RPE conditions for farmers in low- and middle-income countries and tropical climates, the RPE available from rural community markets, which in this study was found to include the robber mask made of woven fabric, cotton mask, and bandana, are alternative RPE for protection against insecticides. The price of these RPE ranged between 0.49 and 4.96 USD, which is cheaper than the half facepiece respirator by approximately 25–256 times. So, the farmers can afford to buy these cheaper items and they are easily available. Furthermore, these RPE items are made of cotton fabric which can absorb a large amount of water when compared with synthetic fibers, allowing the absorbance of sweat, reducing worker discomfort [40]. Due to these characteristics, wearers feel more comfortable and accept wearing these items of RPE while handling pesticides.

The main limitation is that this study was conducted in the laboratory. Co-factors in field conditions should be considered, such as the fit factor of the RPE, inward leakage of the RPE, and skin temperature when wearing RPE at work [27,43,44]. Variations in human breathing, both inhalation and exhalation, could also create differences as the pump used in this study is representative only of inhalation. Therefore, evaluation of the filtration efficiency including co-factors in field conditions needs to be carried out in further studies. This study was conducted using only insecticides, which are widely used in agriculture. Studies using other pesticides, especially herbicides and fungicides, should be conducted to extend the usability and transferability of the data.

## 5. Conclusions

The wearing of a surgical mask was the least effective method as regards protection against insecticide exposure; therefore, it is unsuitable for wearing when handling pesticides. The half facepiece respirator was the first choice to reduce exposure to insecticides, the second choice being the organic vapor respirator. However, the RPE available from rural community markets, which included the robber mask made with woven fabric, cotton mask, and bandana, might be alternative RPE for protection against insecticides in low- and middle-income countries, and tropical climate conditions. To improve the health and life of farmers, public health strategies should involve enhancing the knowledge around the selection of suitable RPE to reduce exposure to damaging pesticides.

## Figures and Tables

**Table 1:** Descriptions and images of various respiratory protective equipment (RPE).

Type of RPE		Description
Surgical mask	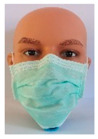	-a standard surgical mask available from pharmacies -made of non-woven material (4 layers), has a nose bridge strip -disposable (single use) -thickness 0.557 ± 0.005 mm -price 0.17–0.33 USD
Sun hat	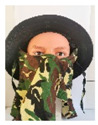	-made of cotton woven fabric -available from rural markets in Thailand -reusable -thickness 0.306 ± 0.004 mm -price 2.65–2.98 USD
Robber mask (woven fabric)	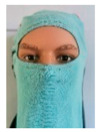	-made of cotton woven fabric -available from rural markets in Thailand -reusable -thickness 0.555 ± 0.006 mm -price 2.15–2.48 USD
Robber mask (knitted fabric)	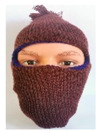	-made of cotton knitted fabric -available from rural markets in Thailand -reusable -thickness 1.508 ± 0.452 mm -price 1.49–1.98 USD
Activated carbon mask	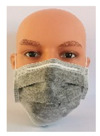	-a standard surgical mask available from pharmacies -made of non-woven material (4 layers), has a nose bridge strip -disposable (single use) -thickness 0.487 ± 0.001 mm -price 0.17–0.33 USD
cotton mask	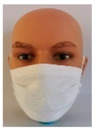	-made of cotton woven fabric -available from markets in Thailand -reusable -thickness 0.577 ± 0.002 mm -price 0.49–0.66 USD
Bandana	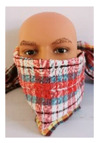	-made of cotton woven fabric -farmers usually fold into quadruple layers when using -available from markets in Thailand -reusable -thickness 1.467 ± 0.009 mm -price 3.96–4.96 USD
Organic vapor respirator without valve	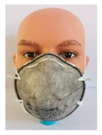	-a certified disposal respirator by 3M (8247) -meets NIOSH 42 CFR84 (R95) -made of non-woven material -has a nose bridge strip -5 layers: polyester layer, polypropylene layer, activated carbon layer, and 2 polypropylene layers -available from 3M distributor -disposable (single use) -price 3.14 USD
Organic vapor respirator with valve	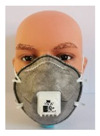	-a certified disposable respirator with valve by 3M (9913V) -meets the requirements of Australian/ New Zealand Standard (AS/NZS 1716:2012) (GP1) -made of non-woven material -has a nose bridge strip -5 layers: polyester layer, polypropylene layer, activated carbon layer, and 2 polypropylene layers -available from 3M distributor -disposable (single use) -price 3.27 USD
Half facepiece respirator	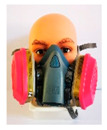	-half facepiece respirator 7502 by 3M (7502) -cartridge 60926 (multi-gas/vapor cartridge) -meets NIOSH 42 CFR84 (P100) -face seal, inhalation and exhalation valve made with silicone rubber -available from 3M distributor -reusable -price 125.50 USD

Note: AS/NZS = Australian/New Zealand Standard; NIOSH = The National Institute for Occupational Safety and Health.

**Table 2:** Quality control of test insecticides.

Types of Insecticides	LOD (μg)	LOQ (μg)	%Recovery
Chlorpyrifos	0.0020	0.0200	110.6
Profenofos	0.0050	0.0500	89.7
Omethoate	0.1000	0.5000	97.9
Diazinon	0.0010	0.0100	98.4
Cypermethrin	0.0010	0.0100	110.6
Deltamethrin	0.0010	0.0100	87.6

**Table 3:** Insecticide concentrations (mg/mL) in the pad from mannequin with and without RPE.

Insecticide		No Mask	Surgical Mask	Sun Hat	Robber Mask (Woven)	Robber Mask (Knitting)	Activated Carbon Mask	Cotton Mask	Bandana	Respirator-No Valve	Respirator-Valve	Half Facepiece Respirator
Chlorpyrifos	Mean	0.581	0.261	0.136	0.207	0.114	0.149	0.101	0.0997	0.109	0.0971	0.012
	SD.	0.14	0.097	0.088	0.143	0.033	0.060	0.032	0.033	0.042	0.043	0.006
	Median	0.53	0.24	0.124	0.161	0.106	0.143	0.099	0.092	0.111	0.107	0.012
Profenofos	Mean	0.405	0.258	0.146	0.101	0.114	0.065	0.075	0.084	0.057	0.047	0.009
	SD.	0.192	0.059	0.111	0.126	0.039	0.028	0.043	0.044	0.026	0.021	0.005
	Median	0.393	0.250	0.116	0.048	0.100	0.070	0.058	0.069	0.045	0.054	0.011
Omethoate	Mean	2.22	1.500	0.606	0.668	0.515	0.234	0.514	0.432	0.524	0.272	0.109
	SD.	1.03	0.287	0.610	0.976	0.465	0.040	0.544	0.223	0.379	0.257	0.106
	Median	2.4	1.620	0.328	0.252	0.381	0.222	0.258	0.326	0.294	0.218	0.079
Diazinon	Mean	2.37	1.310	0.455	0.890	0.438	0.700	0.490	0.392	0.471	0.389	0.028
	SD.	0.461	0.776	0.250	0.323	0.178	0.310	0.109	0.074	0.222	0.187	0.017
	Median	2.34	1.420	0.496	0.834	0.484	0.636	0.532	0.396	0.452	0.402	0.027
Cypermethrin	Mean	0.523	0.203	0.122	0.052	0.105	0.025	0.057	0.046	0.031	0.019	0.009
	SD.	0.322	0.043	0.096	0.073	0.027	0.016	0.038	0.030	0.017	0.010	0.005
	Median	0.487	0.202	0.100	0.024	0.103	0.027	0.044	0.040	0.021	0.016	0.010
Deltamethrin	Mean	0.0349	0.0127	0.0073	0.0028	0.0072	0.0026	0.0037	0.0026	0.0017	0.0015	0.0011
	SD.	0.0208	0.0028	0.0058	0.0032	0.0019	0.0008	0.0024	0.0012	0.0007	0.0005	0.0012
	Median	0.034	0.0142	0.0060	0.0016	0.0073	0.0025	0.0031	0.0025	0.0013	0.0012	0.0009

## Data Availability

Not applicable.
